# Distribution of yellow fever vectors in a disease-free area of Northeast Brazil

**DOI:** 10.1590/0037-8682-0111-2025

**Published:** 2026-02-02

**Authors:** Roseli La Corte, Francielma Santos Bittencourt, José Rodrigo Santos Silva, Lázaro Santana Santos, Ricardo Marcelo Geraldi, David Campos Andrade, Luciane Moreno Storti de Melo

**Affiliations:** 1 Universidade Federal de Sergipe, Programa de Pós-Graduação em Biologia Parasitária, São Cristóvão, SE, Brasil.; 2 Universidade Federal de Sergipe, Departamento de Morfologia, São Cristóvão, SE, Brasil.; 3 Universidade Federal de Sergipe, Departamento de Estatística e Ciências Atuariais, São Cristóvão, SE, Brasil.; 4 Universidade de São Paulo, Faculdade de Saúde Pública, São Paulo, SP, Brasil.; 5 Universidade Federal de Sergipe, Departamento de Biologia, São Cristóvão, SE, Brasil.

**Keywords:** Arboviruses, Vector ecology, Entomological surveillance, Epizootics

## Abstract

**Background:**

Owing to the recent spread of the yellow fever virus in Brazil and imminent risk of its establishment in previously disease-free areas in the northeast of the country, epidemiological surveillance actions are necessary, including knowledge of non-human primate populations and the vectors that inhabit risk areas. The objective of this study was to evaluate the movement of sylvatic and urban yellow fever vectors between an Atlantic Forest fragment and its surrounding areas.

**Methods:**

The study site was the Mata do Junco Wildlife Refuge Conservation Unit (CU) in Capela, Sergipe, Brazil. Immatures were collected using ovitraps in the forested area and in peridomestic environments surrounding the CU.

**Results:**

Fourteen species were recorded, six of which were epidemiologically relevant. The main vectors of yellow fever in Brazil were present in the CU. *Aedes albopictus* (Skuse, 1894) was the most abundant species and was present in all studied environments. *Aedes aegypti* (Linnaeus, 1762) was not collected from forested areas. Among the native species, *Haemagogus leucocelaenus* (Dyar & Shannon, 1924) and *Haemagogus janthinomys* (Dyar, 1921) were collected only in the forested area of the CU, whereas *Haemagogus spegazzinii* (Brètes, 1921) was collected both in the forest and in the area around the CU*.*

**Conclusion:**

Exotic species circulate between wild and urban areas for feeding and oviposition, but circulation to the urban area is limited among native species.

## INTRODUCTION

The last major urban yellow fever outbreak occurred between 1928 and 1929 in Brazil, with the final urban case reported in 1942^1^. Since then, cases of the sylvatic form continued to occur primarily in the Amazon and Central-West regions. However, during the first two decades of this century, several outbreaks of the virus were recorded outside the Amazon region, culminating in one of the most substantial events in the history of sylvatic yellow fever in Brazil. Between 2016 and 2019, the disease spread through previously unaffected areas in the southeastern states, where more than 4,000 epizootics and 2,237 human cases of yellow fever were confirmed^2^ ^-^ ^4^. In the Northeast region, during this reemergence event, yellow fever epizootics were confirmed in the state of Bahia, although no human cases were recorded^5^.

Although non-human primates of the genus *Alouatta* are the most affected by the yellow fever virus, during the recent outbreak, many marmosets (*Callithrix* spp.) were diagnosed with the virus, including animals from urban areas^6^ ^-^ ^8^. Marmosets are common in the peripheral areas of several cities, urban parks, and residential condominiums^9^ ^-^ ^12^, where they function as epidemiological sentinels but are also victims of the disease.

Mosquito species of the genera *Haemagogus* and *Sabethes,* widely distributed across tropical regions, act as vectors of the yellow fever virus in wild environments^13^ ^-^ ^15^. In Brazil, *Haemagogus leucocelaenus* (Dyar & Shannon, 1924) and *Haemagogus janthinomys* (Dyar, 1921) are considered the primary vectors of the virus^16^ and are abundant in the Atlantic Forest, particularly during the rainy season^17^ ^,^ ^18^. The exotic species *Aedes aegypti* (Linnaeus, 1762) and *Aedes albopictus* (Skuse, 1894) remain widely distributed in Brazil despite intensive control efforts and are regarded as epidemiological links with the potential to facilitate the re-establishment of the urban transmition^19^ ^-^ ^21^. In the context of yellow fever reemergence, understanding the Culicidae fauna is crucial for surveillance, particularly in regions where Culicidae surveys are sporadic, such as the Northeast of Brazil^22^ ^-^ ^24^. A lack of comprehensive data can undermine estimates of species distribution and consequently the assessment of arbovirus transmission risk.

This study aimed to survey the fauna of sylvatic and urban yellow fever vectors in a Conservation Unit (CU) in the Atlantic Forest and its surroundings in the state of Sergipe, in the light of the findings of a study by Li et al. (2022)^24^, which suggested that the state of Sergipe is not suitable for *Hg. janthinomys* and only its western part is suitable for *Hg. leucocelaenus,* despite the presence of Atlantic Forest fragments. Given the proximity between forested and urban areas, and the potential movement of yellow fever vectors between sylvatic and urban environments, we also tested the hypothesis that primary vector species move between these settings in search of oviposition sites, which may facilitate the transfer of the yellow fever virus between anthropogenic and natural areas.

## METHODS

### Study area

**FIGURE 1:**
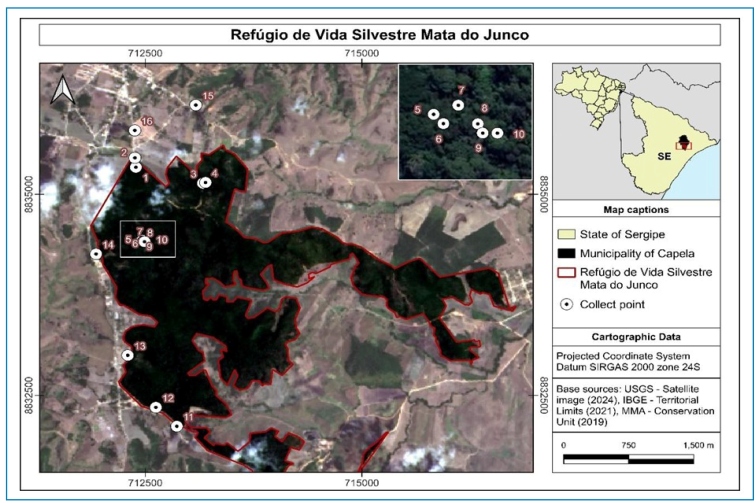
Location of the Mata do Junco Wildlife Refuge Conservation Unit, and the 16 collection points in Capela, Sergipe, Brazil.

The study area comprised the *Refúgio de Vida Silvestre Mata do Junco* CU located in the municipality of Capela in the state of Sergipe, 67 km from the capital Aracaju (10°30′35″ S and 37°03′17″ W) (Figure 1). The climate is classified as equatorial dry summer (As), according to the updated Köppen-Geiger climate classification^25^, with an average annual temperature of 24.9 °C and accumulated annual precipitation of 1372 mm. The rainy season runs from April to July, with monthly average above 100 mm; the dry season runs from October to January, with monthly rainfall average below 50 mm. The CU is composed of fragmented forest patches resulting from intensive logging prior to its consolidation and is classified as a sub-deciduous Atlantic Forest, island type^26^. The area covers 894.90 ha, of which only 498.61 ha are covered by vegetation, characterized as secondary forest under regeneration^27^. According to floristic studies conducted in the area, the average canopy height was 12 m, with some trees reaching up to 20 m, which is considered low according to Atlantic Forest standards. Fabaceae had the highest tree species richness, followed by Myrtaceae. The most abundant species belonged to the families Lecythidaceae, Myrtaceae, Sapotaceae, Fabaceae, and Melastomataceae. The canopy structure ranges from closed areas with lianas, epiphytes, and climbers, to more open areas^28^ ^-^ ^30^. 

The CU was created with the aim of protecting fragments of the Atlantic Forest, the endangered species *Callicebus coimbrai* (Kobayashi & Langguth, 1999) (Coimbra Filho's titi monkey), and preserving the source of Rio Lagartixo, which supplies water to the municipality of Capela^26^ ^,^ ^28^. In addition to *C. coimbrai, Callithrix jacchus* (Linnaeus, 1762) (marmosets) and, occasionally, *Sapajus* sp. (capuchin monkeys) are found in the area^31^ ^,^ ^32^. The CU provides habitats for sylvatic vectors and is located in close to anthropized areas, where artificial containers suitable for oviposition are available.

### Sample collection and processing

Ovitraps were used to collect eggs and occasionally larvae and pupae^33^. Each ovitrap consisted of a black plastic container with a wide opening that was partially filled with water. A fiberboard paddle was positioned as an oviposition substrate with a rough surface facing outward and fixed vertically inside the container using a metal clip. Immatures were collected monthly between March 2019 and February 2020. Traps were installed at 10 points within the CU and six points in its surroundings. Within the CU, four points were located in anthropized areas (Figure 1, 1-4), close to the reserve administration building and water collection station, in places where human activities frequently occur (hereinafter referred to as the open area); the remaining six points within the CU were located in forested areas (Figure 1, 5-10) (hereinafter referred to as the forest area). The six additional points were established in rural areas surrounding the CU (Figure 1, 11-16). At every location, two traps were installed at different vertical strata: one at 1 m from ground level and the other at 3 m, targeting both female mosquitoes that oviposit at low heights and acrodendrophilic species; thus, a total of 32 traps were installed. The maximum height was defined based on the average height of the bifurcations of the main trunks (potential sites for cavity formation) of trees located in rural areas surrounding the forest, where tree height was generally lower than that in the forested area. Ovitraps were installed by securing them to trees and bamboo trunks within the forest and to fruit trees, beneath roof eaves or on water tank platforms in rural areas, positioned near potential mosquito habitats and protected from rain. The paddles and remaining water in the traps were checked once a month and replaced. 

The collected contents were sent to the insectarium of the Tropical Entomology and Parasitology Laboratory (LEPaT) at the Federal University of Sergipe. Specimens collected in the pupal form were placed in cages until adults emerged. When present, *Toxorhynchites* larvae were placed into individual vials to prevent predation. After drying, the paddles were examined under a stereoscopic microscope, and all eggs present were counted regardless of whether they had hatched. Paddles were maintained on a drying rack for one week and subsequently immersed in trays containing 400 mL of mineral water and 0.01 g of fry food granules until larval hatching. The larvae were reared to the fourth-instar stage, after which they were either sacrificed or individually isolated for the collection of larval e pupal exuviae. Immediately after emergence, adults were placed in a freezer for 20 min, transferred to microtubes containing silica, and stored in a refrigerator until use.

### Mosquito identification

Taxonomic identification was carried out following the keys available^14^ ^,^ ^34^ ^-^ ^37^. The genus was abbreviated according to Reinert (2001)^38^. For the genus *Aedes*, the old taxonomic classification was maintained as recommended by Wilkerson et al. (2015)^39^.

The identification of some species was also confirmed at the molecular level by DNA barcoding. Depending on the availability, one to five specimens of each species were processed. DNA was extracted from whole adult specimens using the PureLink® Viral RNA/DNA Mini Kit (Life Technologies, Carlsbad, CA, USA). The primers LCOI490 and HCO2198^40^ were used to amplify an approximately 658 bp fragment located at the 5′ end of the Cytochrome Oxidase subunit I sequence, which was trimmed to 609 bp. Both the PCR and sequencing reaction were run using the Phire Tissue Direct PCR Master Mix (Thermo Scientific™, Anjou, QC, CA), according to the manufacturer's instructions. PCR products were electrophoresed on a 2% agarose gel stained with Diamond Nucleic Acid Dye (Promega Corporation). The products were sequenced in both directions with the same set of primers and analyzed in an ABI 3130 DNA Analyzer (PE Applied Biosystems, Warrington, UK); the quality of the chromatograms was verified visually in Chromas v2.6.5 (Technelysium Pty Ltd, Brisbane, AU). Homologies, insertions/deletions, and frameshifts were identified using MUSCLE v3.8.31^41^. The sequences obtained in FASTA format were aligned using ClustalW^42^. The sequence from each specimen was compared to barcode sequences from GenBank using BLAST. The Cytochrome Oxidase subunit I sequences were deposited in the National Center for Biotechnology Information nucleotide sequence database. The degree of divergence was determined using the genetic distance matrix in MEGA v6.0^43^. DNA amplification was unsuccessful for specimens preserved in naphthalene.

### Statistical analysis

The richness was estimated based on the number of species and morphotypes. Species were characterized according to their relative abundance and constancy^44^, categorized as constant (c) when recorded in more than 50% of collections, accessory (a) when recorded in 25-50% of collections, and occasional (t) when recorded in fewer than 25% of collections.

The analyses were performed using R version 3.6.1^45^. Normality of the data was tested using the Shapiro-Wilk statistical test. The correlation between distribution and precipitation was assessed using Spearman’s correlation test. Precipitation data from an Agrometeorological Monitoring System were used^46^. To compare the distribution of Culicidae in terms of collection areas, a multinomial test was used, considering the difference in the number of collection points between the areas. Species abundance was compared according to collection height using a binomial test. For all analyses, a significance level of 0.05 was considered. 

### Ethical approval

This study did not involve human experimentation. The umbrella project entitled “Investigation of the Circulation of Yellow Fever Virus, Hematozoa and Gastrointestinal Endoparasites in Neotropical Primates in the State of Sergipe” was approved by the UFS Animal Research Ethics Committee, the Chico Mendes Biodiversity Institute (ICMBio), Sergipe State Secretariat for Urban Development and Sustainability, and registered with the National System for the Management of Genetic Heritage and Associated Traditional Knowledge (SISGEN).

## RESULTS

Richness of 14 species was observed, distributed across six genera: *Aedes, Culex, Haemagogus*, *Limatus, Toxorhynchites*, and *Wyeomyia*. Among the identified species, *Ae. aegypti*, *Ae. albopictus*, *Hg. janthinomys, Hg. leucocelaenus*, and *Haemagogus spegazzinii* (Brètes, 1921) are considered of medical importance^36^. Several species showed homology with sequences deposited in GenBank; however, some showed a divergence greater than 3% (Table 1). The species of the genus *Toxorhynchites* was morphologically identified as *Toxorhynchites theobaldi* (Dyar & Knab, 1906), using the available key^35^; however, because of the divergence found with the sequence of Mexican origin deposited in GenBank (KY782655; identity 94.90%, coverage 99.00%, and a genetic distance of 5.9%), in contrast to the morphological homology with another species collected by our group in the State of Bahia (MF537259; identity 99.84%, coverage 89%, and a genetic distance of 0.1%), we chose to maintain the species as *Toxorhynchites* sp.^23^.

**TABLE 1:** Species and accession codes of Cytochrome Oxidase subunit I gene sequences deposited in GenBank.

Species	Access number	(n)	Closest species in GenBank	Closest species access number	Identity % (Coverage%)/Genetic distance %
*Aedes aegypti*	PP264736,	3	*Ae. aegypti*	MN298998.1	99.84 (100)/0.0
	PP372844-				
	PP372845				
*Aedes albopictus*	PP372846-	2	*Ae. albopictus*	MZ501561.1	100.00 (100)/0.1
	PP372847				
*Culex originator*	PP372853	1	*Cx originator*	MF172307.1	95.71 (99)/4.6
*Haemagogus janthinomys*	PP372859	1	*Hg. janthinomys*	MK575481.1	99.09 (100)/0.9
*Haemagogus leucocelaenus*	PP372854-	3	*Hg. leucocelaenus*	MH118163.1	99.40 (99)/0.6
	PP372856				
*Toxorhynchites* sp.	PP372848-	5	*Tx. sp.*	MF537259	99.84 (89)/0.1
	PP372852				
*Wyeomyia arthrostigma*	PP372857	1	*Wy. arthrostigma*	MF172429.1	95.50 (100)/3.7

**FIGURE 2:**
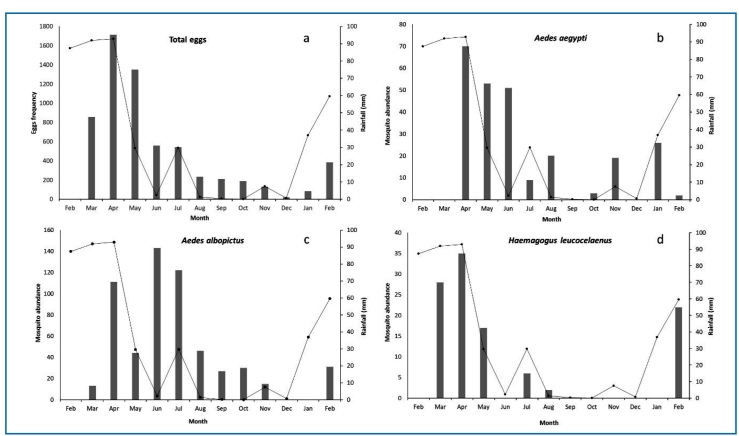
Accumulated precipitation in the 30 d prior to collection and the number of eggs collected by month in areas of Mata do Junco, in the municipality of Capela, Sergipe, from 2019 to 2020; **a** - total eggs; **b** - *Aedes albopictus*; **c** - *Aedes aegypti*; **d** - *Haemagogus leucocelaenus.*

A total of 6,277 eggs, 2,508 larvae, and 42 pupae were collected from the ovitraps. The greatest abundance was observed in April and May 2019, consistently decreasing afterwards before increasing again in February of the following year, following an increase in the precipitation rate in January (Figure 2a ). The pattern observed in the distribution of the number of eggs over time showed a moderate correlation with the accumulated precipitation of the previous 30 d (ρ = 0.56; p = 0.0585) but a strong correlation with the accumulated precipitation in the month prior to collection (ρ = 0.79; p = 0.0002) or in the 60 d prior to collection (ρ = 0.72; p = 0.0082).

After laboratory rearing, 1,355 specimens were identified: 1,203 were adults and 152 were fourth-stage larvae. The most abundant species was *Ae. albopictus* (42.9%), followed by *Ae. aegypti* (18.6%) and *Hg. leucocelaenus* (8.1%). *Haemagogus janthinomys* and *Hg. spegazzinii* had abundances of 1.3% and 0.93%, respectively (Table 2). Of the three most common species, *Ae. albopictus* (ρ = 0.36; p = 0.23) *and Ae. aegypti* (ρ = 0.28; p = 0.36) did not show a correlation with precipitation in the month prior to collection; however, *Hg. leucocelaenus* (ρ = 0.81; p = 0.0012) showed a strong correlation with rainfall (Figure 2b-d ).

**TABLE 2:** Abundance and distribution of Culicidae species captured in ovitraps at different heights and sites in Mata do Junco Wildlife Refuge, Capela, Sergipe, Brazil, 2019-2020.

Species	Height			Trap site				C	N (%)
	1 m	3 m	P-value heights	Refuge Open area	Refuge Forest area	Rural area	Local P-value		
	N (%)	N (%)		N (%)	N (%)	N (%)			
*Aedes aegypti*	181 (71.8)	71 (28.2)	<0.0001	11 (4.4)	0 (0.0)	241 (95.6)	<0.0001	c	252 (18.6)
*Aedes albopictus*	327 (56.2)	255 (43.8)	0.0032	267 (45.9)	64 (11.0)	251 (43.1)	<0.0001	c	582 (42.9)
*Aedes fulvithorax*	30 (41.7)	42 (58.3)	0.1945	45 (62.5)	26 (36.1)	1 (1.4)	<0.0001	c	72 (5.3)
*Aedes terrens*	38 (63.3)	22 (36.7)	0.0518	37 (61.7)	23 (38.3)	0 (0.0)	<0.0001	c	60 (4.4)
*Culex originator*	30 (34.8)	56 (65.2)	0.0066	10 (11.6)	76 (88.4)	0 (0.0)	<0.0001	c	86 (6.3)
*Culex* (*Mcx*) Group: *pleuristriatus*	0 (0.0)	5 (100.0)	-	0 (0.0)	0 (0.0)	5 (100)	-	t	5 (0.4)
*Haemagogus janthinomys*	7 (41.2)	10 (58.8)	0.6291	11 (64.7)	6 (35.3)	0 (0.0)	<0.0001	a	17 (1.3)
*Haemagogus leucocelaenus*	54 (49.1)	56 (50.9)	0.9241	26 (23.6)	84 (76.4)	0 (0.0)	<0.0001	c	110 (8.1)
*Haemagogus spegazzinii*	4 (33.3)	8 (66.7)	0.3877	5 (41.7)	0 (0.0)	7 (58.3)	0.0112	t	12 (0.9)
*Limatus durhamii*	60 (61.2)	38 (38.8)	0.0333	33 (33.7)	41 (41.8)	24 (24.5)	0.0169	c	98 (7.2)
*Limatus flavisetosus*	8 (88.9)	1 (11.1)	0.0390	0 (0.0)	9 (100.0)	0 (0.0)	-	t	9 (0.7)
*Limatus* sp. (damaged)	1 (100.0)	0 (0.0)	-	0 (0.0)	1 (100.0)	0 (0.0)	-		1 (0.1)
*Toxorhynchites* sp.	16 (47.1)	18 (59.9)	0.8642	6 (17.6)	24 (70.6)	4 (11.8)	<0.0001	c	34 (2.5)
*Wyeomyia arthrostigma*	10 (66.7)	5 (33.3)	0.3018	0 (0.0)	15 (100.0)	0 (0.0)	-	a	15 (1.1)
*Wyeomyia* (*Pho*) *pilicauda*	1 (50.0)	1 (50.0)	1.0000	2 (100.0)	0 (0.0)	0 (0.0)		t	2 (0.2)
TOTAL	767 (56.6)	588 (43.4)	< 0.0001	453 (33.43)	369 (27.2)	533 (39.3)			1355 (100.0)

**C:** Constancy; **a:** accessory; **c:** constant; **t:** accidental; **n:** number of mosquitoes.

The number of mosquitoes identified in traps installed at a height of 1 m (56.6%) was significantly higher than the number identified in traps at 3 m (43.3%) (p < 0.0001). However, considering the total number of eggs collected, the abundance was significantly higher at 3 m (53.5%) (p < 0.0001). Of the 15 species captured, *Culex (Microculex)* group *pleuristriatus* was only captured at heights above 3 m. *Aedes aegypti*, *Ae. albopictus*, and *Limatus durhamii* (Theobald, 1901) were significantly more abundant at 1 m, whereas *Culex originator* (Gordon & Evans, 1922) was most abundant at 3 m. There were no statistically significant differences between heights for any *Haemagogus* species.

The relative abundance was significantly higher in the rural area surrounding the CU, whereas species richness was greater in the forest. *Aedes aegypti* was statistically more abundant in rural sites and was absent from the forest, occurring only in open areas of the CU. *Aedes albopictus* was recorded in all three sampling environments and was most abundant in open CU sites. *Haemagogus leucocelaenus* and *Hg. janthinomys* were absent from rural collections and were statistically more abundant in forest and open CU environments respectively. *Haemagogus spegazzinii* occurred in open and rural sites, where it was significantly more abundant.

Among the species of medical importance, *Ae. aegypti, Ae. albopictus*, and *Hg. leucocelaenus* were constant in the traps, whereas *Hg. janthinomys* was an accessory species. Of the other species, *Aedes fulvithorax* (Lutz, 1904), *Aedes terrens* (Walker, 1956), *C. originator, Li. durhamii*, and *Toxorhynchites* sp. were constant species; *Wyeomyia arthrostigma* (Lutz, 1905) was considered accessory; and *Culex (Mcx)* group *pleuristriatus, Hg. spegazzinii, Limatus flavisetosus* (Oliveira Castro, 1935), and *Wyeomyia* (*Pho.*) *pilicauda* (Root, 1928) were occasional species (Table 2).

## DISCUSSION

To date, there is no evidence of reemergence of the yellow fever virus in the forest areas of the State of Sergipe. The last reported outbreak in Sergipe and neighboring states dates back to 1926^47^. Regarding vector distribution, Li et al. (2022)^24^ estimated that the presence of *Hg. janthinomys* and *Hg. leucocelaenus* was unlikely; however, these primary sylvatic vectors of yellow fever virus were recorded in the present study*,* in addition to the exotic species *Ae. aegypti.* Other species that are experimentally competent or have been found naturally infected were also recorded, including *Ae. albopictus* and *Hg.* spegazzinii^5^ ^,^ ^48^ ^-^ ^50^.

Taken together, these results, combined with the presence of non-human primates, indicate high local receptivity to the sylvatic yellow fever transmission. The CU area studied here comprises a small fragment of the Atlantic Forest located close to urbanized areas with limited vegetation cover (Figure 1); however, it represents an important refuge for wildlife, including mosquitoes^51^. This scenario is likely to be repeated across Atlantic Forest remnants along the coastal region of northeastern Brazil^52^.

In Brazil, *Hg. janthinomys* has been found from the North to the Southeast, whereas *Hg. leucocelaenus* extends from the North to the South of the country^13^ ^,^ ^15^. The Culicidae fauna of northeastern Brazil remains poorly sampled; there are only occasional and non-systematic records of the distribution of *Hg. leucocelaenus* and *Hg. janthinomys,* except for the Atlantic Forest of Bahia, where *Hg. janthinomys* has been documented^14^ ^,^ ^23^ ^,^ ^53^. In this survey, *Hg. leucocelaenus* and *Hg. janthinomys* were recorded exclusively in forested areas, although previous studies indicate that both species can disperse long distances-up to 5.7 km and 11.5 km, respectively^54^-and that *Hg. leucocelaenus* may occur in anthropogenic environments, including peri-urban areas^55^. The forested area of Capela presents slightly lower mean annual air temperature and relative humidity (23.7 °C and 89.5%) than clearing areas (24.3 °C and 82.6%)^56^, conditions that may favor sylvatic species and may account for their restriction to the forested habitats in the presence of vertebrate hosts.

As studies targeting adult mosquitoes have successfully recorded *Haemagogus* species in degraded areas^57^, it is possible that these species forage for blood meals in anthropic environments but return to forest for shelter and oviposition. This behavior may explain their absence in ovitraps from rural areas in our study; however, it does not preclude their circulation in areas surrounding the forest. Only *Hg. spegazzinii*, a common species in northeastern Brazil occurring in both the Caatinga and Atlantic Forest biomes^5^ ^,^ ^58^, was successfully collected as immature in rural areas. Although its vector competence is unknown, *Hg. spegazzinii* has previously been reported as naturally infected^48^ ^,^ ^59^. Its wide distribution, potential vector competence, and ability to circulate between wild and anthropogenic environments suggest that this species may play an important role in arboviruses transmission in northeastern Brazil.

Typically, epizootics and epidemics of diseases transmitted by mosquitoes are associated with the beginning of the rainy season, when vector population densities are higher^60^. In this study, the abundance of immature forms in ovitraps was correlated with precipitation, although there was an artificial supply of water to the ovitraps. This additional water availability appeared to favor the exotic species *Ae. albopictus* and *Ae. aegypti*, whereas native species followed a precipitation pattern (Figure 2).

In this study, *Ae. albopictus* was the most abundant species and was recorded at all three collection sites. This species has a wide geographic distribution in Brazil^61^ ^-^ ^65^, and in Sergipe, it was first detected in 2011, followed by its gradual spread throughout the state^65^. Dispersal studies have shown that *Ae. albopictus* females can disperse long distances within a few days, from wild to modified environments^66^. This dispersal capacity, together with ecological adaptability, enables the species to occupy both wild and urban environments. Its ability to feed on a wide range of mammals and its high susceptibility to infection make *Ae. albopictus* a potential risk factor for yellow fever transmission in urban areas^67^ ^-^ ^69^.

Recent yellow fever outbreaks have been reported in Brazil in forested areas near urban centers, including locations without previous evidence of viral activity or established vaccination policies^16^ ^,^ ^69^ ^,^ ^70^. In the Northeast region, yellow fever vaccination began to be recommended in the second half of 2020; however, implementation was hindered by the prioritization of COVID-19 (SARS-CoV-2) vaccination. As a result, vaccination coverage remains low in many municipalities across the region, increasing vulnerability to human cases of this arbovirus.

Despite their acrodendrophilic behavior, *Haemagogus* species were recorded at both heights in this study, suggesting that the availability of suitable habitats may outweigh height in determining oviposition sites. However, this finding should be interpreted with caution, as trap placement was limited to a maximum height of 3 m. While no significant differences were reported between 1 m and 3 m by Silva-Inácio et al. (2020)⁵⁹, Dias et al. (2023b) ^71^ documented greater *Haemagogus* abundance at higher strata (6-8 m).

Study limitations include discrepancies between the total of immature collected and the number ultimately available for identification. Mortality during rearing may have affected the representativeness of the sample, as more fragile species could have been disproportionately impacted by laboratory handling, leading to an underestimation of their frequencies. The exclusive use of a single trap type (ovitraps) may also have limited the detection of species that do not use this container, for example, species of the genus *Sabethes* ^72^.

Recent yellow fever epizootics have resulted in population declines of approximately 30% in golden lion tamarins (*Leontopithecus)* and 10-26% in the muriquis (*Brachyteles hypoxanthus* (Kuhl, 1920)) in Southeast Brazil^73^ ^,^ ^74^. If the virus were introduced into the CU Mata do Junco, it could place the endangered *C. coimbrai* at critical risk of extinction^75^. In addition to *C. coimbrai*, *C. jacchus* also occurs in the Mata do Junco*.* During the 2016 and 2017 outbreaks in Brazil, several epizootics were reported among marmosets. Because marmoset groups move between forest fragments and urban and peri-urban environments, they may act as a link in the yellow fever transmission cycle and facilitate virus spread in these areas^5^ ^-^ ^8^. 

The presence, abundance and constancy of sylvatic (*Hg. leucocelaenus*, *Hg. janthinomys*, and *Hg. spegazzinii*) and urban (*Ae. aegypti* and *Ae. albopictus*) yellow fever vectors across forested, rural, and peridomestic environments, suggest mosquito movement between areas and the potential interaction between sylvatic and anthropogenic transmission cycles. In the context of non-human primates, including endangered species, low vaccination coverage, and proximity to areas with reported epizootics, these findings highlight the importance of sustained entomological and epizootic surveillance in this CU and in other Atlantic Forest remnants in northeastern Brazil. 

## Data Availability

Data-in-article: Research data is available in the body of the document (Table 1 and 2, Figure 2).
